# Abstracts

**DOI:** 10.1155/S1463924686000019

**Published:** 1986


Journal of Automatic Chemistry/*Journal of Clinical Laboratory Automation*, Volume 8, Number 1 (January–March 1986), pages 1–5

### Abstracts

### Page 6

### The introduction of microcomputers into the undergraduate teaching laboratory

H. Elliott *et al.*

The problems of providing chemistry students with experience of computer-linked instrumentation as part of their studies is analysed. The approach to these problems adopted by one college is described. The experience provided to students is based on data handling by free-standing microcomputers and the use of existing instrumentation interfaced to microcomputers to illustrate data acquisition and instrumental control.

The difficulties of using commercial general-purpose data-handling packages in limited-time student practical periods is described.

The development of student experiments involving instrumentation linked to Apple IIe and BBC Model B microcomputers, using commercial interface systems, is described. The Apple IIe has been linked to a range of instruments using the Adalab interface and commercial software. The BBC Model B interfacing has been based on the Super Rexagan general-purpose interface, using software written in-house. The two approaches to interfacing existing instrumentation are compared, and the differing problems of implementation within the constraints of the rigidly timetabled student practical period discussed.

The success of the work in simulating modern, dedicated computer-based instrumentation is portrayed. The effect of the work in producing improved student experimentation and in establishing student attitudes to the use of the computer in experimental work is discussed.

### L'introduction de microordinateurs dans le laboratoire d'enseignement secondaire

H. Elliott *et al.*

Les problèmes qui surviennent lors de la confrontation d'étudiants en chimie avec des instruments connectés à des ordinateurs comme part de leurs études sont analysés. Le traitement de ces problèmes adoptés par un certain collège est décrit. L'expérience a permis aux étudiants de traiter des données avec des microordinateurs séparés et l'utilisation d'instruments existants connectés à des microordinateurs pour illustrer l'acquisition de données et le contrôle des instruments.

Les difficultés survenues lors de l'utilisation de logiciels commerciaux d'utilité générale pendant le temps limité du cours sont discutées.

Le développement d'expériences d'étudiants incluant l'instrumentation connectée à un Apple IIe et des microordinateurs BBC modèle B utilisant des systèmes d'interfaces commerciaux est décrit. Le Apple IIe a été connecté à une série d'instruments utilisant l'interface Adalab et un logiciel commercial. L'interface du BBC modèle B a été basée sur l'interface Super Rexagan générale utilisant un logiciel développé au collège. Les deux variantes pour interfacer des instruments existants sont comparées et les problèmes différents dûs aux contraintes d'un horaire d'étudiants rigide sont décrits.

Le succès du travail de simulation d'instruments modernes avec ordinateurs dédicacés est présenté. L'effet de ce travail pour produire les meilleures conditions d'expérimentation pour les étudiants et pour établir des attitudes d'étudiants envers l'utilisation de l'ordinateur pour le travail expérimental est discuté.

### Einführung des Mikrorechners ins Studenten-Praktikum

H. Elliott *et al.*

Die Probleme werden analysiert, die dadurch entstehen, dass Chemie-Studenten als Teil ihres Lehrganges Erfahrungen mit rechnerverbundener Instrumentierung sammeln können. Der Ansatz, den ein College zur Lösung dieser Probleme macht, wird beschrieben. Die Erfahrungen, die Studenten machen können, basieren auf der Datenverarbeitung mit frei verfügbaren Mikrorechnern und der Verwendung vorhandener Instrumente, die mit Rechnern gekoppelt sind, zur Illustration der Datenerfassung und Instrumentensteuerung.

Die Schwierigkeit der Verwendung allgemeiner, kommerzieller Datenverarbeitungspakete in zeitlich begrenzten Praktiken wird beschrieben.

Die Entwicklung von Studentenversuchen mit Instrumenten, die mit Apple IIe und BBC, Modell B, Rechnern durch kommerzielle Interface-Systeme gekoppelt sind, wird beschrieben. Der Apple IIe wurde mit einer Reihe von Instrumenten mit Hilfe des Adalab Interface und kommerzieller Software gekoppelt. Der BBC, Modell B, wurde mit Hilfe des allgemein verwendbaren Super Rexagan Interface und selbst geschriebener Software gekoppelt. Diese beiden Ansätze wurden miteinander verglichen, und die unterschiedliche Problematik aus der Sicht der beschränkt verfügbaren Praktikumszeit der Studenten wird beschrieben.

Der Erfolg der Arbeit bei der Simulation moderner Instrumentierung mit fest eingesetztem Rechner wird dargestellt. Die Wirkung der Arbeit durch verbesserte Studentenversuche und durch die Etablierung eines Studentenverhaltens zum Einsatz von Rechner bei experimenteller Arbeit wird beschrieben.

### Page 12

### Design and evaluation of an image-dissector-based derivative spectrophotometer

T. A. Nevius and H. L. Pardue

This paper describes the construction of an image-dissector-based derivative spectrophotometer. The derivatives are computed directly from intensity versus wavelength data recorded in a conventional scanning mode. The quantitative results indicate that this approach can provide information that is similar in quality to that obtained by electronic or wavelength – modulated derivative spectrophotometers. Potential applicability of this derivative spectrophotometer to analytical problems was illustrated by the examination of mixtures of polynuclear aromatic hydrocarbons. Data from the spectrophotometer indicate that two-component mixtures can be resolved with limits of detection (95% confidence level) ranging from about 0.2 g/ml for a 16 ms integration time to about 3 μg/ml for a 512 ms integration time.

**Construction et évaluation d'un spectrophotomètre dérivatif basé sur dissection d'images**

T. A. Nevius and H. L. Pardue

Cette publication décrit la construction d'un spectrophotomètre dérivatif basé sur dissection d'images. Les dérivées sont calculées directement à partir des données d'intensité vers la longueur d'ondes enregistrées dans un mode de balayage conventionnel. Les résultats quantitatifs indiquent que cette façon de faire procure des informations de qualité similaire à celle obtenue par spectrophotomètres dérivatifs avec modulation électronique ou de longueur d'onde. Les possibilités d'application de ce spectro-photomètre dérivatif aux problèmes analytiques ont été illustrées par l'étude de mélanges d'hydrocarbures aromatiques polynucléaires. Les données du spectrophotomètre indiquent que des mélanges de deux composantes peuvent être résolus avec des limites de détection (niveau de confiance 95%) allant de 0.2 g/ml avec un temps d'intégration de 16 ms jusqu'à environ 3 μg/ml pour un temps d'intégration de 512 ms.

**Konstruktion und Beurteilung eines Bildabschnitt-basierten Ableitungs-Spektrophotometers**
T. A. Nevius and H. L. Pardue

Diese Arbeit beschreibt die Konstruktion eines 'Image-Dissector-Based' Ableitungs-Spektrophotometer. Die Ableitungen werden direkt aus den konventionell aufgenommenen Intensitäts/Wellenlängen-Daten berechnet. Die quantitativen Daten weisen darauf hin, dass dieser Ansatz Informationen generiert, die von ähnlicher Qualität sind wie diejenigen von elektronisch oder Wellenlängen-modulierten Ableitungs-Spektrophotometern. Die mögliche Anwendung dieses Ableitungs-Spektrophotometers auf analytische Probleme wird mit einer Untersuchung von Mischungen von polynuklearen aromatischen Kohlenwasserstoffen illustriert. Daten des Spektrophotometers zeigen, das 2-Komponenten Mischungen mit Detektionsgrenzen (95% Vertrauensniveau) von 0.2 g/ml bis 16 ms Integrationszeit bis zu 3 μg/ml bei 512 ms Integrationszeit aufgelöst werden können.

---

### Page 18

**Multicentre evaluation of the IL Densiscan**
P. A. Bonini *et al.*

The characteristics and performance of a new automatic densitometer (IL Densiscan, Instrumentation Laboratory SpA, Italy) has been evaluated. Two laboratories participated: H. S. Raffaele, Milano and Ospedale Civile Stradella, Pavia. The first one evaluated precision and capability of minima detection; the second looked at sensibility and identification criteria of protein fractions.

Reproducibility was very good for serum proteins, lipoproteins and haemoglobins in comparison with a reference instrument; capability of minima detection was excellent. Some problems appeared when protein concentration was very low.

Automatic fraction indentification provides good results for normal patterns; abnormal fractions need optical inspection of the pherogram.

**Evaluation multiple d'un densitomètre automatique IL Densiscan**
P. A. Bonini *et al.*

Les caractéristiques et la performance d'un nouveau densitomètre automatique (IL Densiscan, Instrumentation Laboratory SpA, Italie) ont été évaluées. Deux laboratoires ont participé: H. S. Raffaele, Milan et Ospedale Civile Stradella, Pavie. Le premier a évalué la précision et les limites de détection, le second la sensibilité et les critères d'identification de fractions de protéines.

La reproductibilité était très bonne pour les protéines de sérum, les lipoprotéines et hémoglobines en comparaison avec un instrument de référence; les limites de détection étaient excellentes. Certains problèmes ont apparu lors de concentrations de protéines très basses.

Identification automatique de fractions donne de bons résultats pour mélanges normaux; les fractions abnormales demandent une inspection visuelle du phérogramme.

**Evaluation des IL Densiscan automatischen Densitometers**
P. A. Bonini *et al.*

Die Charakteristiken und Leistungen des neuen automatischen Densitometers (IL Densiscan) wurden ausgewertet. Zwei Laboratorien nahmen daran teil: H. S. Raffaele, Mailand und Ospedale Civile Stradella, Pavia. Das erste Labor evaluierte Präzision und die Fähigkeit der Erfassung von Minima. Das zweite Labor prüfte die Empfindlichkeit und die Kriterien für die Identifikation von Protein-Fraktionen.

Die Reproduzierbarkeit für Serumproteine, Lipoproteine und Hämoglobine war im Vergleich mit einem Referenzinstrument sehr gut. Die Fähigkeit der Erfassung von Minima war ausgezeichnet. Probleme traten auf, wenn die Proteinkonzentration sehr tief war.

Die automatische Fraktionsidentifikation erzielte bei normalem Muster gute Resultate; abnormale Fraktionen erfordern eine optische Inspektion des Pherogramms.

### Page 23

### Automatic detection of the autocorrelation-type measurement error component
K. M. Hangos *et al.*

A new numerical method for the detection of autocorrelation-type measurement error components in measurement processes is described for cases where drift and random measurement error components are also present.

The method was compared with a known graphical method (LAG-1) for the control of liquid sample sequences—a good agreement between the two methods was found.

The authors' method, unlike the graphical method, does not need any personnel decisions during an evaluation, but only requires computation of the sample means and standard deviations. The computation is based on known statistical tests (the t-test and F-test) so it can be automated with microcomputer based laboratory monitoring systems.

This method can be used for automatic validation and quality control of analytical measurements.

### Détection automatique des composantes d'erreurs de mesure du genre autocorrélation
K. M. Hangos *et al.*

Une nouvelle méthode numérique pour la détection des composantes d'erreurs de mesure du genre autocorrélation est décrite pour les processus de mesure qui donnent lieu a des erreurs au hasard ou qui montrent une dérive.

La méthode a été comparée avec la méthode graphique bien connue (LAC-1). Pour une série de contrôle d'échantillons liquides une bonne coïncidence des deux méthodes a été trouvée.

La méthode des auteurs ne nécessite aucunes décisions subjectives pendant l'exploitation, tout au contraire de la méthode graphique. Uniquement le calcul des valeurs moyennes et des écarts types est nécessaire. Le calcul se base sur les testes statistiques connus (t-test et f-test), est donc facile à automatiser sur les calculatrices de laboratoire.

La méthode peut donc être utilisée pour le contrôle de qualité automatique et pour la validation des résultats d'analyse.

### Automatische Detektion von autokorrelationsartigen Messfehlerkomponenten
K. M. Hangos *et al.*

Es wird eine neue Methode für die Detektion von autokorrelationsartigen Messfehlerkomponenten beschrieben für diejenigen Fälle, wo Drift und statistisch zufällige Messfehlerkomponenten auftreten.

Die Methode wurde mit der bekannten grafischen Methode (LAC-1) verglichen. Für Kontrollsequenzen von Flüssigproben wurde eine gute Uebereinstimmung der beiden Methoden gefunden.

Die Methode der Autoren benötigt, im Gegensatz zur grafischen Methode, keine persönlichen Ermessensentscheide während der Auswertung. Erforderlich sind nur die Berechnung der Mittelwerte und Standardabweichungen. Die Berechnung basiert auf bekannten statistischen Testen (t-Test und F-Test), ist also leicht auf Laborrechnern automatisierbar.

Die Methode kann für die automatische Validierung und Qualitätskontrolle von Analysenresultaten verwendet werden.

---

### Page 28

### Treatment of super oxide dismutase assay by a regression method
Ph. Nirde

A set of computer programs are described for the automatic exploitation of the enzymatic assay of super oxide dismutase (E.C. 1.15.1.1.). The programs, which use a least squares estimation and a polynomial curve fitting, allow a precise determination of the amount of enzyme found in a sample. This determination is available for a rate of inhibition in the range 0–100% of the standard curve. In addition, owing to the calculation of the curve equation, neither dilution nor concentration are necessary to make the assay. With the advent of microcomputers in laboratories this program allows a rapid determination and renders the assay more sensitive.

### Résumé
Ph. Nirde

Un programme informatique a été établi pour une exploration automatique du dosage enzymatique de la superoxide dismutase (E.C 1.15.1.1). Le programme utilise la méthode des moindres carrés et l'ajustement polynomial d'une courbe standard. Cette méthode permet une déternation précise de la quantité d'enzyme contenu dans un échantillon biologique. Cette détermination est valable sur tout le domaine de la courbe standard. De plus, grâce au calcul de l'équation de la courbe, aucune dilution ni concentration de l'échantillon n'est nécessaire pour effectuer le dosage. Avec l'apparition des micro-ordinateurs dans les laboratoires ce programme permet à la fois une détermination rapide du dosage ainsi qu'une augmentation de sa sensibilité.

### Anwendung der Regressionsmethode auf dem Superoxid-Dismutase-Test
Ph. Nirde

Es wird ein Satz Computer-Programme für die automatische Auswertung der enzymatischen Analyse von Superoxid-Dismutase beschrieben (E.C. 1.15.11). Die Programme, welche eine Methode der kleinsten Quadrate und eine polynomische Kurvenanpassung benützen, erlauben eine genaue Bestimmung der in der Probe gefundenen Enzymmenge. Diese Bestimmung ist für eine Inhibitions geschwindigkeit im Bereich von 0–100% der Standardkurve verfügbar. Ferner ist wegen der Art der Berechnung der Kurvengleichung weder eine Verdünnung oder Eindickung vor der Analyse notwendig. Mit dem Aufkommen der Mikrocomputer im Labor erlaubt dieses Programm eine rasche Bestimmung und macht die Analyse empfindlicher.

**Page 32**

**Interfacing a Cary 210 spectrophotometer to a Commodore PET 2001 microcomputer**
D. Migneault *et al.*

A Commodore PET microcomputer has been interfaced to control and acquire data from a Cary 210 spectrophotometer. The interface was accomplished from the memory expansion port of the PET through a MCS-6522 Versatile Interface Adapter to the Digital Interface Port of the Cary. A sample benzene vapour spectrum is presented.

**Interface d'un spectrophotomètre Cary 210 à un microordinateur Commodore PET 2001**
D. Migneault *et al.*

Un microordinateur Commodore PET a été interfacé pour contrôler et acquérir des données d'un spectrophotomètre Cary 210. L'interface a été éffectuée à partir de la sortie d'expansion de mémoire du PET à travers un MCS-6522 adapteur versatile d'interface à l'entrée digitale d'interface du Cary. Un spectre de vapeurs de benzène est présenté comme example.

**Kopplung eines Cary 210 Spektrophotometers mit einem Commodore PET 2001 Mikrocomputer**
D. Migneault *et al.*

Ein Commodore PET Mikrocomputer wurde mit einem Cary 210 Sepktrophotometer für Steuerung und Datenerfassung gekoppelt. Die Kopplung wurde zwischen dem Speichererweiterungsanschluss des PET zum Digitaleingang des Cary mit einem MCS-6522 Versatile Interface Adapter bewerkstelligt. Als Beispiel wird das Spektrum von Benzoldampf angeführt.

---

**THE FOLLOWING ABSTRACTS SHOULD HAVE BEEN PUBLISHED IN OUR LAST ISSUE, WE APOLOGIZE FOR THE DELAY:**

**Page 173**

**Evaluation du dosage du cholestérol sur le seralizer Ames au laboratoire et en salle d'hôpital**
L. M. Nelson *et al.*

Le seralizer Ames a été évalué au laboratoire et dans l'unité de soins coronaires afin de déterminer si les caractéristiques de performance permettaient de faire le dosage du cholestérol près du malade avec les advantages cliniques que cela présente. Dans l'évaluation du laboratoire, utilisant des critères objectifs modernes d'acceptabilité, la linéarité était statistiquement satisfaisante tandis que l'imprécision et l'inexactitude n'étaient pas satisfaisantes. La performance réalisée par trois jeunes médecins était nettement inférieure à celle obtenue au laboratoire. L'instrument est donc considéré peu convenable pour l'utilisation au laboratoire et en salle d'hôpital pour le dosage du cholestérol, à l'exception éventuellement pour une détection de l'hypercholestérolaemia.

**Beurteilung des Ames Serlayzer für die Cholesterin-Bestimmung im Labor und auf der Station**
L. M. Nelson *et al.*

Der Ames Seralyzer wurde im Labor und auf der Herzkrankenstation geprüft um zu bestimmen, ob das Leistungsspektrum die Durchführung von Cholesterin-Analysen nahe beim Patienten, was klinisch vorteilhaft ist, erlaubt. Unter Verwendung moderner objektiver Kriterien für die Akzeptanz erwies sich bei der Beurteilung im Labor die Linearität als statistisch genügend, aber Präzision und Genauigkeit als ungenügend. Die Resultate, die drei jüngere Aerzte erzielten, waren signifikant schlechter als die Laborergebnisse. Das Instrument wird deshalb als ungeeignet für die Cholesterin-Bestimmung inner- und ausserhalb des Labors betrachtet. Eine Ausnahme davon bildet vielleicht die Detektion der Hypercholesterolaemie an vorderster Front.

---

**Page 192**

**Micro-ordinateurs dans un laboratoire de contrôle des eaux**
M. P. Bertenshaw and K. C. Wheatstone

Les autorités responsables pour les eaux doivent analyser un grand nombre d'échantillons de tous les domains du cycle hydrologique afin de contrôler et de conserver les standards de qualité de l'eau requis pour de l'eau potable, pour les rivières et les éffluants. Par example, Severn-Trent Water Authority, une parmi 10 en Angleterre et Wales, effectue environ 2.5 millions de déterminations chimiques et bactériologiques par an. Le système de traitement de données doit donc procurer la base pour l'automation à l'intérieur d'un laboratoire de contrôle des eaux, mais également prévoir les possibilités de transférer des résultats hors du laboratoire. Cette publication présente des domaines dans lesquels des microordinateurs ont été utilisés pour faire le pont entre des instruments commerciaux et un système de traitement de données de laboratoire. Les avantages de la réception automatique des données et les domaines pour le développement futur sont discutés.

**Mikrocomputer in einem amtlichen Wasserkontrolllabor**
M. P. Bertenshaw and K. C. Wheatstone

Amtliche Wasserkontrollorgane müssen eine grosse Zahl von Proben aus allen Gebieten der hydrologischen Zyklen analysieren, um damit den erforderlichen Standard der Wasserqualität in der Trinkwasserversorgung, in Flüssen und im Abwasser überwachen und aufrecht erhalten zu können. Zum Beispiel führt die Severn-Trent Water Authority, eine von zehn in England und Wales jährlich ungefähr 2,5 Millionen chemische und bakteriologische Bestimmungen durch. Das Datenverarbeitungssystem muss demzufolge auf eine Basis für die Automation im Labor ausgerichtet sein und auch für den Bericht von Resultaten ausserhalb des Labors vorgesehen sein. Die vor vorliegende Arbeit zeigt, wie Mikrocomputer für die Brücke zwischen kommerziell erhältlicher Instrumentierung zur automatisierten und manuellen Wasseranalyse und einem Labordatenverarbeitungssystem eingesetzt wurden. Die Vorteile der automatischen Datenerfassung und Gebiete der Weiterentwicklung werden diskutiert.

---

**Page 201**

**Chromatographie à haute performance à échange de ions pour acides aminés dans des fluides**

**biologiques utilisant Chromakon 500 – performance de l'instrument**
L. Cynober *et al.*

La performance analytique d'un système de chromatographie à haute pression pour échange de ions (Kontron Chromakon 500) pour des mesures d'acides aminés dans des fluides biologiques a été examinée. 39 acides aminés et leurs dérivés ont été séparés. Les temps de rétention étaient constants avec un coefficient de variation entre 0.1% à 1.3%. La réaction était linéaire jusqu'à au moins 1250 μmol/l pour tous les acides aminés. Les limites de détection étaient en général de 5 μmol/l. Les écarts-type d'un dosage étaient inférieurs à 5% excepté pour la phénylalanine 8.3%. La reproductibilité moyenne était de 10.2%. Le Chromakon 500 procure donc des résultats satisfaisants pour tous les acides aminés en 170 minutes; ce qui inclut le temps de régénération.

**Hochleistungs-Ionenaustausch-Chromatografie von Aminosäuren in biologischen Flüssigkeiten mit dem Chromakon 500 – Leistungsspektrum des Gerätes**
L. Cynober *et al.*

Es wurde die analytische Leistung des Hochdruck-Ionenaustausch-Chromatographen (Kontron Chromakon 500) für Aminosäuren-Messungen in biologischen Flüssigkeiten getestet. Die Retentionszeiten blieben konstant, mit Variationskoeffizienten von 0.1 bis 1.3%. Die Reaktion war bis zu mindestens 1250 μmol/l für alle Aminosäuren linear. Die Detektionsgrenzen waren generell 5 μmol/l. Die Variationskoeffizienten von Wiederholbarkeitsanalysen betrug weniger als 5%, mit Ausnahme von Phenylalanin (8.3%). Die mittlere Reproduzierbarkeit betrug 10.2%. Der Chromakon 500 scheint somit für alle Aminosäuren genügende Resultate rasch, nämlich in 170 Minuten inklusive Regenerationszeit, zu liefern.

### PITTSBURGH 1986

The arrangements for this year's Pittsburgh Conference and Exposition on Analytical Chemistry and Applied Spectroscopy (Atlantic City Convention Center, Atlantic City, New Jersey, USA: 10–14 March) are now being finalized. The organizers have sent *JAC* the following updates on previously published information:

*Technical programme*

An expanded number of technical sessions for 1986 will be presented Monday 10 March to Noon Friday 14 March 1986. All technical sessions will be held in the Atlantic City Convention Center and the two hotels adjacent to the Convention Center. The technical program will include 30 symposia and approximately 1100 contributed papers and poster displays.

*Exposition of modern laboratory equipment*

Over 700 of the major companies dealing in spectroscopic and general analytical instrumentation/equipment will be exhibiting their latest products in more than 1900 booths. The exposition will be open Monday from 9.00 a.m. to 5.30 p.m. and Tuesday through Thursday from 8.30 a.m. to 5.30 p.m.

*Continuing education*

This year the short course topic will be electrochemistry. The chemical and electrical foundations will be laid for discussions of important applications such as chemical sensors, voltammetry and electrochemical detection. The three instructors, Professors Buck, Heineman and Kissinger are active in research, as well as being independently associated with small businesses and clinical and analytical service laboratories that use their working electrochemical devices. The short course will be presented Friday afternoon and Saturday morning.

Mini-courses will be taught on 14 March.

*Special awards*

The Spectroscopy Society of Pittsburgh is pleased to announce that Dr Tomas B. Hirschfeld has been selected to receive the *1986 Pittsburgh Spectroscopy Award.* Dr Hirschfeld, currently of the Lawrence Livermore Laboratory, is being recognized for his outstanding contributions to FTIR as well as for his many notable achievements in a number of other scientific areas including, but not limited to, NIR, ATR, diffuse reflectance spectroscopy, fluorescence spectroscopy, Raman spectroscopy, fibre optics, microsensors, and robotics. SSP Chairlady Jane H. Judd will present the award to Dr Hirschfeld during a special symposium scheduled for Wednesday 12 March.

Professor Gary M. Hieftje will receive the *1986 Pittsburgh Analytical Chemistry Award* in recognition of his work in flame and plasma chemistry and physics. The award is sponsored by the Society for Analytical Chemists of Pittsburgh. Professor Hieftje will receive the award on Tuesday morning, 11 March 1986.

Professor Gerhard Schomburg will receive the *1986 Dal Nogare Award* for his outstanding contributions in the fields of gas and liquid chromatography. The award is sponsored by the Chromatography Forum of the Delaware Valley. Professor Schomburg will receive the award on Tuesday afternoon, 11 March 1986.

Professor Fred C. Anson will receive the third *Charles N. Reilley Award* for his fundamental work in adsorbates, modified electrodes and electrocatalysis. The award is sponsored by Bioanalytical Systems, Inc., and given by the Society for Electroanalytical Chemistry. Professor Anson will receive the award on Tuesday afternoon, 11 March 1986.

Dr Abraham Savitsy and Dr Joseph J. Barrett are the joint recipients of the *1986 Williams-Wright Award* sponsored by the Coblentz Society. Dr Savitsky is being honoured for his numerous contributions to spectroscopy, especially computer methods. Dr Barrett is being honoured for the development and application of Raman spectroscopy for industrial use. Drs Savitsky and Barrett will receive the award on Thursday afternoon, 13 March 1986.

*Registration forms from George L. Vassilaros, Registration Chairman, 12 Federal Drive, Suite 322, Pittsburgh, Pennsylvania 15235, USA.*

